# A Novel Small Molecule GDNF Receptor RET Agonist, BT13, Promotes Neurite Growth from Sensory Neurons *in Vitro* and Attenuates Experimental Neuropathy in the Rat

**DOI:** 10.3389/fphar.2017.00365

**Published:** 2017-06-21

**Authors:** Yulia A. Sidorova, Maxim M. Bespalov, Agnes W. Wong, Oleg Kambur, Viljami Jokinen, Tuomas O. Lilius, Ilida Suleymanova, Gunnar Karelson, Pekka V. Rauhala, Mati Karelson, Peregrine B. Osborne, Janet R. Keast, Eija A. Kalso, Mart Saarma

**Affiliations:** ^1^Laboratory of Molecular Neuroscience, Institute of Biotechnology, University of HelsinkiHelsinki, Finland; ^2^Department of Anatomy and Neuroscience, The University of MelbourneMelbourne, VIC, Australia; ^3^Department of Pharmacology, Faculty of Medicine, University of HelsinkiHelsinki, Finland; ^4^Molcode Ltd.Tartu, Estonia; ^5^Department of Molecular Technology, Institute of Chemistry, University of TartuTartu, Estonia; ^6^Pain Clinic, Department of Anesthesiology, Intensive Care and Pain Medicine, University of Helsinki and Helsinki University HospitalHelsinki, Finland

**Keywords:** neuropathic pain, glial cell line-derived neurotrophic factor (GDNF), GDNF family ligands (GFLs), artemin (ARTN), small molecule RET agonist, GFL mimetic, GDNF family receptor α (GFRα), receptor tyrosine kinase RET

## Abstract

Neuropathic pain caused by nerve damage is a common and severe class of chronic pain. Disease-modifying clinical therapies are needed as current treatments typically provide only symptomatic relief; show varying clinical efficacy; and most have significant adverse effects. One approach is targeting either neurotrophic factors or their receptors that normalize sensory neuron function and stimulate regeneration after nerve damage. Two candidate targets are glial cell line-derived neurotrophic factor (GDNF) and artemin (ARTN), as these GDNF family ligands (GFLs) show efficacy in animal models of neuropathic pain (Boucher et al., 2000; Gardell et al., 2003; Wang et al., 2008, 2014). As these protein ligands have poor drug-like properties and are expensive to produce for clinical use, we screened 18,400 drug-like compounds to develop small molecules that act similarly to GFLs (GDNF mimetics). This screening identified BT13 as a compound that selectively targeted GFL receptor RET to activate downstream signaling cascades. BT13 was similar to NGF and ARTN in selectively promoting neurite outgrowth from the peptidergic class of adult sensory neurons in culture, but was opposite to ARTN in causing neurite elongation without affecting initiation. When administered after spinal nerve ligation in a rat model of neuropathic pain, 20 and 25 mg/kg of BT13 decreased mechanical hypersensitivity and normalized expression of sensory neuron markers in dorsal root ganglia. In control rats, BT13 had no effect on baseline mechanical or thermal sensitivity, motor coordination, or weight gain. Thus, small molecule BT13 selectively activates RET and offers opportunities for developing novel disease-modifying medications to treat neuropathic pain.

## Introduction

Neuropathic pain—defined by the International Association for the Study of Pain as “pain caused by a lesion or disease of the somatosensory nervous system”— severely impairs quality of life and is a significant socio-economic burden. The estimated prevalence of neuropathic pain ranges from 1–2 to 9.8% (Yawn et al., 2009; Smith and Torrance, 2012; van Hecke et al., 2014; IASP, 2015) and is expected to grow due to the aging population and increases in diseases that damage peripheral sensory neurons. Causes of neuropathic pain include traumatic nerve injury, disease (e.g., diabetes, cancer), infection (HIV), or exposure to certain drugs—particularly chemotherapeutic agents (Ossipov, 2011; Finnerup et al., 2015). Additional risk factors include age, female gender, genetic factors, and surgical interventions (Shiri et al., 2007; IASP, 2015; Momi et al., 2015). Existing treatments for neuropathic pain are not effective in a proportion of patients, and are often accompanied by intolerable adverse effects (Finnerup et al., 2015). Importantly, the currently available drugs are not disease-modifying, i.e., they do not “cure” the disease.

Neurotrophic factors are proteins that can modulate neuropathic pain states by targeting sensory neurons to affect functions that include survival, axon regeneration, and neuronal excitability. For example, the neurotrophins nerve growth factor (NGF) and brain-derived neurotrophic factor (BDNF) increase pain sensation (Obata and Noguchi, 2006; McKelvey et al., 2013); and their sequestration with antibodies (Li et al., 2008; Lane et al., 2010; Brown et al., 2013; Gimbel et al., 2014; Kan et al., 2016) or disruption of NGF signaling (Owolabi et al., 1999; Winston et al., 2003; Ugolini et al., 2007) reduces pain in patients or in animal models, but does not restore damaged neurons.

Systemic administration of the glial cell line-derived neurotrophic factor (GDNF) family ligand (GFL) artemin (ARTN) can have protective effects in animal models of experimental nerve-injury, as measured by increased regeneration and functional recovery, and transient reductions in hyperalgesia following nerve-injury (Boucher et al., 2000; Gardell et al., 2003; Harvey et al., 2010; Takasu et al., 2011; Hedstrom et al., 2014; Wang et al., 2014). Recent Phase 1 clinical trials (Rolan et al., 2015; Okkerse et al., 2016) suggest ARTN is now being assessed as a potential clinical treatment for peripheral nerve injury and a disease-modifying therapy for neuropathic pain. However, clinical translation could prove to be limited by the poor drug-like properties of ARTN and other GFL proteins—which include low bioavailability, poor diffusion in tissues (Piltonen et al., 2009), and being expensive and laborious to produce to clinical standards. The possibility of adverse side effects is also raised by evidence of ARTN and GDNF having pronociceptive or proalgesic actions in some *in vitro* and *in vivo* assays (Elitt et al., 2006; Bogen et al., 2008; Hendrich et al., 2012; Lippoldt et al., 2013; Ikeda-Miyagawa et al., 2015) or when used at high doses in Phase I clinical trials (Rolan et al., 2015; Okkerse et al., 2016). These properties of the endogenous GFL protein ligands suggest that successful clinical translation could be facilitated by developing small molecule GFL mimetics that retain neurotrophic activity but show a more selective pharmacological profile and superior drug-like properties.

All four GFLs—GDNF, ARTN, neurturin (NRTN), and persephin (PSPN)—signal through the transmembrane receptor tyrosine kinase RET. The binding specificity is provided by a cell surface-bound GPI-anchored GDNF family receptor α (GFRα): GDNF preferentially binds to GFRα1, NRTN to GFRα2, ARTN to GFRα3, and PSPN to GFRα4 (Airaksinen and Saarma, 2002; Sidorova et al., 2010). Ligand binding to GFRα/RET leads to autophosphorylation of RET kinase domains and subsequent activation of multiple intracellular signaling pathways including Akt, MAPK-Erk, Src, and JNK cascades (Airaksinen and Saarma, 2002). At least two alternative GDNF receptors are known: neural adhesion molecule (NCAM; Paratcha et al., 2003) and heparan sulfate proteoglycan syndecan-3 (Bespalov et al., 2011), which mediate some biological effects of GDNF.

The first candidate small molecule GFL mimetic, XIB4035, was described by a Japanese group (Tokugawa et al., 2003). However, it was later shown to increase the activity of GDNF or ARTN rather than activate GFL receptors. Nevertheless, XIB4035 alleviated experimental diabetic neuropathy in rodents (Hedstrom et al., 2014).

Here, we report on using high-throughput screening (HTS) to identify a GFL mimetic that can elicit a biological response, independently from GFL proteins, by directly targeting RET. This molecule named BT13 potently and selectively activates RET and its downstream intracellular signaling cascades in immortalized cells, promotes neurite outgrowth from cultured dorsal root ganglia (DRG) sensory neurons *in vitro*, alleviates mechanical hypersensitivity and reverses injury-induced changes in protein expression in DRG neurons in animals with the spinal nerve ligation (SNL)-induced experimental neuropathy.

## Materials and methods

### Reagents and animals

#### Proteins

GDNF, ARTN, and BDNF were obtained from PeproTech Ltd. NGF was purchased from Promega, PeproTech, or Sigma-Aldrich.

#### Plasmids

Full-length human *GFR*α*1* cDNA subcloned in pcDNA6 (Invitrogene), full-length human *GFR*α*3* cDNA (Wang et al., 2006), full-length human *RET* (long isoform) in pCR3.1 (Invitrogene; Runeberg-Roos et al., 2007), and PathDetect Elk-1 system (Stratagene) to detect MAPK activation were used in this study.

#### Cell lines

MG87RET murine fibroblasts were stably transfected with RET proto-oncogene (Eketjäll et al., 1999). MG87TrkA, MG87TrkB murine fibroblasts were stably transfected with TrkA or TrkB receptor tyrosine kinase (Glass et al., 1991; Ip et al., 1993). Reporter gene systems used to detect MAPK activation were: MG87RET stably transfected with PathDetect Elk-1 and GFRα1, GFRα3 or empty vector (Sidorova et al., 2010), MG87TrkB stably transfected with PathDetect Elk-1 (Sidorova et al., 2010), or MG87TrkA stably transfected with PathDetect Elk-1.

#### Animals

Animal experiments were conducted in accordance to local and European regulation and guided by 3R principles. The provincial government of Southern Finland approved the study concept (Etelä-Suomen aluehallintovirasto, Hämeenlinna, Finland, ESAVI/5684/04.10.03/2011) for locomotor activity and acute pain sensitivity. Primary cultures of DRG neurons were prepared from adult (6–8 weeks) female Sprague-Dawley rats supplied by the School of Biomedical Sciences Animal Facility at the University of Melbourne. These animals were used in procedures and experiments approved by the Animal Ethics Committee of the University of Melbourne in accordance with National Health and Medical Research Council of Australia guidelines.

Acute pain sensitivity and locomotor activity were tested on male Sprague-Dawley rats (Scanbur, Harlan, Netherlands), weighing 100–125 g, at the University of Helsinki. Experiments in animal models of neuropathic pain were ordered from Psychogenics Inc. (USA) and performed on male Sprague-Dawley rats (100–125 g) from Harlan (Indianapolis, IN, USA). In all cases during the acclimatization period animals were housed in groups (3–4/cage) at ambient temperature of 20–25°C. During the course of the study 12 h light/dark cycles were maintained, with water and standard laboratory chow provided *ad libitum*.

### Procedures using animals

#### Spinal nerve ligation (Chung's) model of experimental neuropathy

Experimental neuropathy in rats was induced by ligation of left L5 and L6 spinal nerves (Kim and Chung, 1992). Before surgery, rats were tested for baseline sensitivity to mechanical stimuli using von Frey filaments and animals with paw withdrawal thresholds (PWT) below 12 were excluded from the study. Rats were subsequently balanced and assigned to treatment groups based on their baseline PWT values (11–12 animals per group). Surgery was performed under isoflurane anesthesia. All rats received an analgesic (buprenorphine, 0.05 mg/kg, subcutaneously) immediately before and 6 h after surgery. Each rat was monitored until it was awake and moving freely around the recovery chamber. Animals were then single-housed for the duration of the study.

#### Treatments

Animals received repeated subcutaneous injections of BT13 (5–25 mg/kg, dissolved in sesame oil containing 5% DMSO), vehicle (sesame oil containing 5% DMSO), or ARTN (0.5 mg/kg, dissolved in saline containing 5% DMSO) on post-surgical day 1, 3, 5, 8, 10, and 12 in the volume of 5 ml/kg. Responsiveness of animals with neuropathic pain to standard analgesics was confirmed by using a single dose of gabapentin (100 mg/kg dissolved in water, in the volume 1 ml/kg) given orally on the 12th day post-surgery 1 h before the behavioral tests. Rats began their treatment regimen (except gabapentin) 1 h post-surgery. In characterization of acute effects, 25 mg/kg of BT13 was used, and administration followed that of neuropathic testing. On post-surgical day 12 substances were administered 1 h prior to behavioral studies. The administration schedules were based on a published study of ARTN protein (Gardell et al., 2003) to facilitate comparison of the outcomes. Thresholds of ipsilateral paws were expressed as a percentage of the contralateral paw thresholds and used in comparisons between the treatment groups. Animals whose thresholds differed more than 2 *SD* from the mean of the group (*n* = 1–2/group) were considered outliers and excluded from further analyses. Thus, in each treatment group there were 9–11 animals.

#### Behavioral tests

The experiments were performed in a randomized and blinded fashion. Both injections and measurements were blinded. In rats with experimental neuropathy, sensitivity to mechanical stimuli was determined using von Frey filaments on the 12th day post-surgery. In healthy animals, acute nociception and motor coordination were assessed using acetone, tail flick, hot plate, paw pressure, and rotarod tests (Ugo Basile, Comerio, Italy, device models 37360, 35100, 37215, and 47700, respectively) in the indicated order. Baseline nociceptive latencies were measured on the day of the experiment, 1 h before the administration of the drugs. Nociceptive responses were re-assessed at 1.5, 3, 24, and 48 h after the first injection, as well as on the 12th day of the experiment, before and 1.5 h after the last injection. Timeframe of behavioral assessment is indicated in **Figures 4B–F**. To avoid tissue damage suitable cut-offs were set in all experiments.

To measure sensitivity to thermal stimuli either an infrared light was directed to the middle third of the tail of a constrained animal or the rat was put on the hot plate adjusted to 52°C (±0.2°C). Latency time before the flick of the tail (tail flick test) or brisk shaking or licking of the hind paw (hot plate test) was assessed. To assess cold allodynia, the acetone test was used. A drop of acetone was applied to the plantar surface of the hind paw twice, with 5 min intervals between the measurements, alternating paws between stimulations. Lifting, sniffing, flicking, shaking, licking, or guarding of the paw as well as startle and jumping were recorded and scored according to the magnitude of the response. To measure sensitivity to mechanical stimuli in the paw pressure test the hind paw of the rat was placed under a pivot and the force applied to the paw was linearly increased. Pressure required to elicit withdrawal of the paw (von Frey filament), or vocalization or brisk shaking of the hind limb (paw pressure test) was determined. To assess motor coordination, the time during which the animal was able to stay on the rotating rod (20 rpm) was measured.

#### Pathological analysis of major organs

Upon completion of behavioral experiments animals were terminated and samples from the heart, skeletal muscle, lungs, liver, pancreas, kidneys, spleen, adrenal glands, peripheral nerves (sciatic nerve), and skin with subcutis at the injection site were examined histologically. Microscopic findings were classified with standard pathological nomenclature and severities of findings were graded on a scale of minimal, mild, moderate, or severe. Grades of severity for microscopic findings were subjective; minimal was the least extent discernible and severe was the greatest extent possible. Microscopic findings that are not usually graded were listed as present.

### Luciferase assays

To identify compounds activating GFL receptors and to check their ability to activate intracellular signaling via TrkA and TrkB, we used previously developed reporter-gene based systems (Sidorova et al., 2010). MG87 murine fibroblasts were stably transfected with Pathdetect Elk-1 and one of the following: TrkA, TrkB, RET, GFRα1/RET, GFRα3/RET. The day before the experiment reporter cells were plated into 96- or 384-well cell culture plates (PerkinElmer) at 175,000–200,000 cell/ml density in DMEM, 10% FBS, 100 μg/ml Normocin (Invivogen, Cat# ant-nr-1), 1% DMSO, 15 mM Hepes pH 7.2. The next day compounds or proteins under study were applied to the cells in desirable concentration. The following day the cells were lysed and luciferase activity was measured using a luciferase detection reagent (SteadyGlo, Cat# E2550, or Luciferase assay reagent, Cat# E1501, Promega). The luminescence was measured using a plate luminometer (Micorbeta-2 or TopCount, PerkinElmer). During high throughput screening each compound was tested in a single concentration (5 μM) and a single repeat. Hit confirmation experiments were performed in triplicates. Dose-dependent studies in all reporter cell lines were made in quadruplicates. EC50 was calculated based on the results of at least three independent experiments.

### Phosphorylation assays

#### Sample preparation

MG87RET, MG87TrkA, or MG87TrkB were plated on 35 mm tissue culture dishes 1–2 days before the experiment to achieve 90–95% of confluency of the cells at the day of stimulation with tested substances. If necessary, cells were transfected with 4 μg/well of GFRα1-, GFRα3-, or green fluorescent protein (GFP)-expressing plasmid using Lipofectamine 2000 (Invitrogen) for DNA delivery as described by manufacturer. Before the experiment cells were starved in serum-free DMEM containing 15 mM HEPES, pH 7.2 and 1% DMSO for 4 h and stimulated with BT13 or neurotrophic factors. Then cells were washed once with ice-cold PBS containing 1 mM Na_3_VO_4_ and 1 mM NaF and lysed on ice in 1 ml per well of RIPA-modified buffer (50 mM Tris-HCl, pH 7.4, 150 mM NaCl, 1 mM EDTA, 1% NP-40, 1% TX-100, 10% glycerol, EDTA-free protease inhibitor cocktail (Roche), 1 mM Na_3_VO_4_, 2.5 mg/ml of sodium deoxycholate, 1 mM NaF). Plates were incubated at +4°C on the horizontal shaker for 30 min with vigorous shaking. Afterwards, 80–100 μl aliquot was taken from each well to prepare whole cell lysates. This aliquot was mixed with equal volume of 2x Laemmli loading buffer (4% SDS, 20% glycerol, 10% 2-mercaptoethanol, 0.004% bromphenol blue, 0.125 M Tris HCl, pH 6.8) and boiled for 10 min at 100°C. Remaining cell lysates were used for immunoprecipitation of RET or Trk receptors.

#### RET, TrkB, and TrkA phosphorylation assay

To immunprecepitate RET, TrkA, and TrkB, cell lysates were incubated overnight at +4°C on a round rotator in the presence of 1 μg/ml of goat anti-RET C-20 (Santa Cruz Biotechnology Cat# sc-1290, RRID:AB_631316) or anti-Trk C-14 antibodies (Santa Cruz Biotechnology Cat# sc-11, RRID:AB_632554) and magnetic beads conjugated with protein G (Dynabeads, Thermo Fisher Scientific). Beads were washed three times with TBS with 1% Triton X-100, and bound proteins were eluted by 100 μl of 2x Laemmli loading buffer, resolved on 7.5% SDS-PAGE and then transferred onto a nitrocellulose membrane. Membrane was blocked for 15 min at room temperature with TBS-T (TBS containing 0.15% Tween 20) containing 10% skimmed milk and probed with anti-phosphotyrosine antibodies (Millipore Cat# 05–321, RRID:AB_309678) diluted 1:1,000 in TBS-T with 3% skimmed milk for 2 h at room temperature. The membranes were washed three times for 5 min in TBS-T and incubated in a 1:1,000 solution of secondary anti-mouse antibodies conjugated with HRP (DAKO, Cat# P0447) diluted in TBS-T containing 3% skimmed milk for 45 min at room temperature. Membranes were washed with TBS-T for 4 × 10 min. Stained bands were visualized with ECL reagent or femptoECL reagent (Pierce) using LAS3000 imaging software. To confirm equal loading, membranes were stripped and reprobed with anti-RET C-20 antibodies (1:500) or anti-Trk C-14 antibodies (1:1,000) diluted in TBS-T containing 3% skimmed milk. To detect C-20 we used secondary anti-goat antibodies conjugated with HRP (1:1,500, DAKO, Cat# P0449), to detect C-14 we used anti-rabbit antibodies conjugated with HRP (1:3,000, GE Healthcare Cat# NA934 RRID:AB_772206).

#### Erk1/2 and Akt phosphorylation assays

Whole cell lysates were resolved on 12% SDS-PAGE and transferred to nitrocellulose membranes. Membranes were blocked with TBS-T containing 10% skimmed milk for 10–15 min and probed with anti-phospho-Akt antibodies (Cell Signaling Technology Cat# 9271L RRID:AB_329826) diluted 1:1,000 in TBS-T containing 5% BSA overnight at +4°C; or with anti-phospho-Erk antibodies (Santa Cruz Biotechnology Cat# sc-7383 RRID:AB_627545) diluted 1:500 in TBS-T containing 3% skimmed milk overnight at +4°C. Then anti-phospho-Akt-treated membrane was incubated in the 1:3,000 solution of secondary anti-rabbit antibodies conjugated with HRP and anti-phospho-Erk-treated—with anti-mouse-HRP-conjugated antibodies diluted in TBS-T containing 3% skimmed milk for 45 min at room temperature. Membranes were washed with TBS-T (4 × 10 min). Stained bands were visualized with ECL reagent or femptoECL reagent (Pierce) using LAS3000 imaging program. To confirm equal loading, membranes were stripped and reprobed with anti-GAPDH antibodies (Millipore Cat# MAB374 RRID:AB_2107445) diluted 1:4,000 in TBS-T containing 3% skimmed milk. To detect anti-GAPDH antibody we used secondary anti-mouse antibodies conjugated with HRP (1:1,000).

### Neurite outgrowth from DRG neurons

Cultured lumbar DRG (L3–L5) neurons were prepared using a published method (O'Mullane et al., 2013). After plating onto poly-L-ornithine (5 μg/ml)/laminin (5 μg/ml) coated coverslips for 1 h, DRG neurons were cultured for 24–30 h in fresh Neurobasal A/B27. GFLs, BT13, NGF, and vehicle control were added at the start of this period for a 24–30 h treatment, except when preceded by pre-treatment (1 h) with inhibitors (50 μM LY294002; 2 μM SU6656, 2 μM PD98059 in 0.5% DMSO v/v; Biomol Research Laboratories, Plymouth Meeting, PA, USA). DRG neurons were then fixed (4% paraformaldehyde) and processed for immunocytochemistry with 1:200 Mouse anti-β III tubulin/AlexaFluor 594 (Sigma-Aldrich Cat# T8660 RRID:AB_477590) and 1:5,000 rabbit anti-calcitonin gene-related peptide/AlexaFluor 488 (Sigma-Aldrich Cat# C8198 RRID:AB_259091) and DAPI for nuclear staining (0.5 μg/ml, Molecular Probes). Image analysis of multichannel 12 bit Zeiss .ZVI or .TIF files (captured with a Zeiss Imager.M1/MRm camera/Zen software; Carl Zeiss Australia, North Ryde, NSW, Australia) was then performed by a researcher blinded to the experimental condition. *Neurite initiation* was measured by a single parameter (proportion of neurons with neurites longer than the soma diameter). *Neurite elongation* was measured by batch processing (HCA-Vision software; CSIRO Mathematical and Information Sciences, North Ryde, NSW, Australia) to obtain four parameters—*Root number:* number of primary neurites; *Maximum length:* length of longest neurite; *Branch points:* number of neurite branch points; and *Total length:* sum of neurite lengths (Kalous and Keast, 2010; Kalous et al., 2012). The number of neurons of each immunocytochemical class counted on each coverslip (technical replicates) was 50 for neurite initiation and 20 for neurite elongation. A minimum of three cultures from different rats (biological replicates) were analyzed for each treatment group.

### Immunohistochemistry

DRGs from rats with spinal nerve ligation treated with BT13 or vehicle were embedded in paraffin and sectioned (7 μm thickness). Sections were deparaffinized, subjected to citrate or basic antigen-retrieval procedure and probed with IB4-Alexa 488 [1:200; IB4-Alexa488, Cat# IB4 (isolectin *Griffonia simplicifolia* IB4) RRID:AB_2314664] and antibodies against calcitonin gene-related peptide (CGRP; 1:10,000, Peninsula Laboratory, Cat# T-4032 RRID:AB_2313775), NPY (1:10,000, Peninsula Laboratory, Cat# T-4070.0050 RRID:AB_518504), pErk1/2 (1:300, Cell Signaling, Cat# 4370L RRID:AB_2297462), pS6 (1:300, Cell Signaling, Cat# 4858L RRID:AB_1031194) or RET [1:100, kind gift of Dr. E. Kramer, Center for Molecular Neurobiology, University Medical Center Hamburg-Eppendorf, Hamburg, Germany (Meka et al., 2015)] and corresponding secondary antibodies conjugated with fluorophores (Donkey Anti-Rabbit Alexa Fluor 647 conjugate, Cat# A31573 RRID:AB_162544; Donkey anti-Mouse Alexa Fluor 647 conjugate, Cat# A-31571 RRID:AB_2536181; Goat anti-Guinea Pig, Alexa Fluor 568 conjugate, Cat# A-11075 RRID:AB_2534119, Thermo Fisher Scientific, USA) or horse radish peroxidase (VECTASTAIN Elite ABC-Peroxidase Kit antibody, Vector Laboratories, CA, USA, Cat# PK-6101 RRID:AB_2336820). In the latter case, to reveal staining slides were treated with ABC and DAB staining kits (Vector Laboratories, CA, USA) according to the manufacturer's instructions. Sections were co-stained with guinea pig anti-PGP9.5 antibodies (Abcam Cat# ab10410 RRID:AB_297150) whenever possible. Nuclei were visualized using hematoxylin or DAPI dyes. Sections were mounted in Immu-Mount (Thermo Scientific, Cat#9990402) or Coverquick 2000 (Q PATH, Cat#05547530) mounting media and images were taken with fluorescent (Zeiss Imager M2 Axio, Carl Zeiss, Germany) or bright-field (Olympus BX-61, Olympus, Japan) microscopes. Neurons were counted for two sections per DRG and the number of cell profiles with nuclei positive for each specific marker was normalized to the total number of the neurons (identified by PGP9.5 staining or by morphology) per section. Ret-positive neurons were clustered by size using Matlab software R2014b (Mathworks, USA). Statistical analysis was performed using data from three to six individual animals. To obtain sufficient sample size, the data from animals treated with 20 and 25 mg/kg of BT13 were combined.

### qPCR

Expression of RET mRNA in DRGs of animals with SNL after receiving vehicle, BT13 and ARTN was analyzed by qPCR. RNA isolation and DNA digestion in the samples was performed using NucleoSpin® RNA kit (Macherey-Nagel Inc., Germany) according to the manufacturer's instructions. RNA concentration was measured with NanoDrop (ND 1000, Thermo Scientific, USA). cDNA was synthesized by Maxima H minus reverse transcriptase (Cat# EP0751, Thermo Scientific, USA) as described by the manufacturer in a total volume 20 μl. Fifteen microliters of the mixture containing 100 ng of RNA, 100 pmol of oligo(dT)_15–18_ (Promega, USA; Oligomer, Finland), 0.5 mM dNTP (ThermoScientific, Cat# R0191) were incubated at 65°C for 5 min to remove possible secondary structure and melt GC-rich regions. After cooling on ice, reverse transcriptase buffer (50 mM Tris-HCl, pH 8.3, 75 mM KCl, 3 mM MgCl_2_, 10 mM DTT, final concentrations), 20 U of RiboLock RNase Inhibitor and 100 U of Maxima H Minus Reverse Transcriptase (all from Thermo Scientific, USA) were added to the reaction mixture. Mixture was incubated for 30 min at 50°C and reverse transcriptase was inactivated by heating (85°C, 5 min). qPCR was performed using LightCycler® 480 SYBR Green I Master (Roche Diagnostics) according to the manufacturer instructions in total volume of 10 μl using LightCycler® 480 system (Roche Diagnostics). Reaction mixture contained 0.25 μl of cDNA, 1xSYBRGreen I Master mix, 2.5 pmol of forward (RET: 5′-CTGGGAGCAGCTCAGCAT-3′; Cyclophilin A: 5′-TATCTGCACTGCCAAGACTGAGTG-3′) and reverse (RET: 5′-TGTGGACCCCAGGAAGAC-3′; Cyclophilin A: 5′-CTTCTTGCTGGTCTTGCCATTCC-3′) primers. Relative expression of target genes was determined by 2^−ΔΔ*CT*^. For normalization of the target gene expression the data on expression of house-keeping gene Cyclophilin A were used.

### CEREP screening

The ability of BT13 in 1 μM concentration to interact with the following targets: adenosine receptors A1 (Cat# 0002) and A2B (Cat# 0005); adrenergic receptors alpha 1 (Cat# 0008), alpha 2 (Cat# 0011), and beta 3 (Cat# 3963); cannabinoid receptor CB1 (Cat# 0036); cholecystokinin receptors CK1 (Cat# 0039) and CCK2 (Cat# 0041); dopamine receptors D1 (Cat# 0044) and D2L (Cat# 1405); GABA receptor GABAB (Cat# 0885); histamine receptors H1 (Cat# 0870) and H2 (Cat# 1208); neurokinin receptors NK1 (Cat# 0100), NK2 (Cat# 0102) and NK3 (Cat# 0104); neurotensin receptor (Cat# 0465); opioid receptors delta (Cat# 0114, 2568), kappa (Cat# 1971), and mu (Cat# 0118); vasoactive intestinal peptide receptors (Cat# 1518); serotonin receptors 5HT1A (Cat# 0131); 5-HT1D (Cat# 1974); 5-HT2A (Cat# 0471); 5-HT2C (Cat# 0137, 1003); Ca^2+^ ion channel (Cat# 0162); KATP channel (Cat# 0165); norepinephrine transporter (Cat# 0355); dopamine transporter (Cat# 0052); serotonin transporter (Cat# 0439); monoamine oxidase A (Cat# 0443); catechol-*o*-methyltransferase (Cat# 0457); protein serine/threonine phosphatase PP2A (Cat# 3953); protein tyrosine phosphatase PTPRC (Cat# 3956) was evaluated by CEREP company (CEREP, France, http://www.cerep.fr/Cerep/Utilisateurs/index.asp) using standard company protocols.

### ADME and cytotoxicity studies

ADME profiling was performed by Cyathus Exquirere (Milan, Italy). To assess BT13 pharmacokinetics, 10 mg/kg of BT13 was intravenously injected into nine male Sprague Dawley rats. Plasma and brain samples were collected 1, 3, and 6 h later. Elimination rate constant was determined from measured plasma concentrations after a single IV bolus. Half-life and volume of distribution of BT13 were then calculated using a one-compartment model. Plasma stability of BT13 was assessed after 1 h incubation with human, rat and mouse plasma, using verapamil as an internal standard and lidocaine and M7319 (Sigma) as reference compounds. Metabolic stability of BT13 was measured after 30 min incubation with 0.5 mg/ml of rat liver microsomes (Xenotech) using verapamil as an internal standard and 7-ethoxycoumarin and propranolol as reference standards. In all above-mentioned cases, samples were analyzed by chromatography on an UPLC system integrated with a Premiere XE Triple Quadrupole. To assess cardiac safety K_i_ of BT13 in competition assay with radiolabelled ligand of the hERG (human *Ether-à-go-go-related* gene) [^3^H]-astemizole in crude cell membrane fraction of HEK293 cells stably transfected with the corresponding gene was measured. BT13 toxicity toward HepG2 cells was determined using the MTT assay. Inhibition of the most important drug-metabolizing isoforms of CYP (CYP1A2, CYP2C9, CYP2C19, CYP2D6, and CYP3A4) was measured using a fluorescent assay with a specific substrate for each isoenzyme. The IC_50_ was determined using a four-parameter curve fit. Furafylline, sulfaphenazole, tranylcypromine, quinidine, and ketoconazole were used as reference compounds for CYPs listed above.

### Statistical analysis

All quantitative data were subjected to statistical analysis using GraphPad Prism v6 (La Jolla, CA, USA). All data are presented as mean ± SEM. Significance of the difference between treatment groups was determined by *t*-tests (two independent sample or paired) or ANOVA with Tukey HSD or Dunnett *post-hoc* tests. *P* < 0.05 was considered to be statistically significant.

## Results

### High throughput screening campaign and hit validation

**Figure 1:**
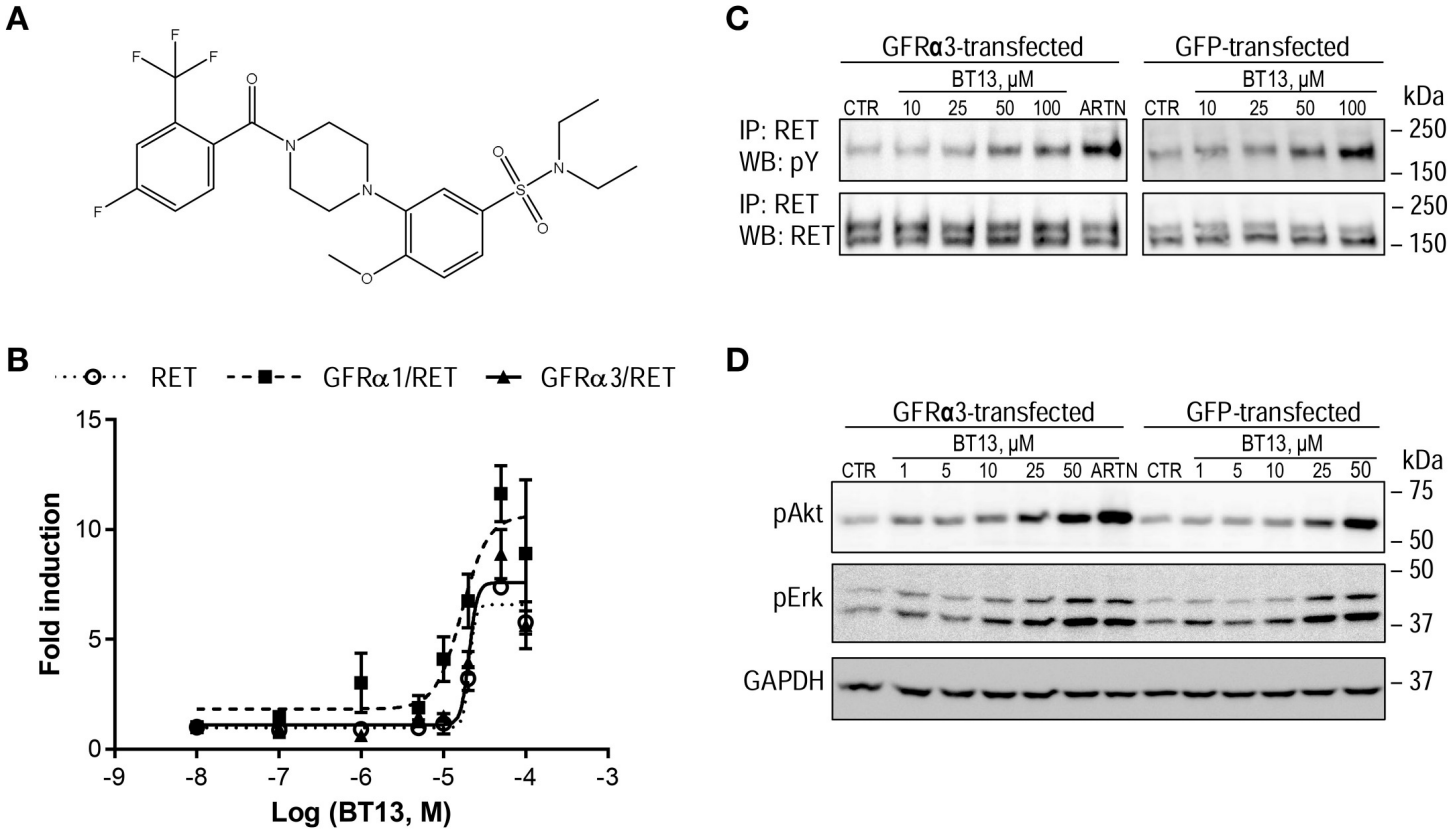
The structure and biological activity of a small molecular weight RET agonist BT13. **(A)** Structure of BT13. **(B)** Fold induction of luciferase activity by BT13 in reporter cell lines expressing GFRα1/RET (GFRα1/RET, dashed line, black squares), GFRα3/RET (GFRα3/RET, solid line, black triangles), or RET (RET, dotted line, empty circles). **(C,D)** Activation of RET phosphorylation **(C)** and RET-dependent intracellular signaling **(D)** by BT13 in cells overexpressing GFRα3/RET (GFRα3-transfected) and GFP/RET (GFP-transfected). Luciferase activity was measured in three independent experiments for each concentration of BT13. GFRα—glycosylphosphatidylinositol (GPI)-anchored GDNF family receptor α; RET, rearranged during transfection; ARTN, artemin; IP, immunoprecipitation; WB, western blotting; pY, phosphotyrosine; pAkt, phosphorylated form of Akt protein; pErk, phosphorylated form of Erk1/2; GAPDH, Glyceraldehyde 3-phosphate dehydrogenase, house-keeping protein, loading control.

To identify small molecule GFL mimetics we selected and screened a diverse set of chemical compounds. A library of 199,082 compounds of >85% purity at >5 mg amount (available at AMRI Inc., Hungary) was virtually filtered through a set of absorption, distribution, metabolism and excretion, and toxicity (ADME-Tox) filters and ranked according to drug likeness index (Xu and Stevenson, 2000). The selected set of 54,000 compounds was further reduced by applying diversity criteria (Papp et al., 2006), and the resulting library of 18,400 compounds was then screened at 5 μM concentration in a cell-based high-throughput assay with a luciferase readout in cells expressing GFRα1 and RET (Sidorova et al., 2010). Forty-three compounds that induced luciferase response by at least three times above the baseline were then tested in luciferase assay in triplicates in 5 μM concentration to confirm their biological activity. Only five molecules were confirmed as hits in these experiments. After validation by re-synthesis and re-testing only one compound that we named BT13 (Figure 1A) reproducibly activated luciferase in the cells expressing GFRα1 and RET.

### HTS hit BT13 selectively activates RET and downstream RET-dependent signaling

BT13 was evaluated further in GFRα1/RET, GFRα3/RET, and RET reporter cell lines at concentrations of 0.1–50 μM. We found that it dose-dependently activated luciferase in all three cell lines with comparable potency (EC_50_ = 17.4, 20.5 and 20.7 μM, respectively) and efficacy (11.6-, 8.9-, and 7.4-fold induction above baseline, respectively; Figure 1B). GDNF in this assay had higher potency (EC_50_ = 1.2 nM) and efficacy (84.9-fold induction above baseline; Sidorova et al., 2010). Next, binding of BT13 to GDNF receptor complexes was evaluated by displacement of ^125^I-GDNF. BT13 (50 μM) reduced ^125^I-GDNF binding by 25 ± 10.2% (*P* = 0.0344, one-tailed *t*-test, *n* = *5*), but testing the higher concentrations of BT13 in this assay was impossible due to poor solubility of BT13 in the binding buffer.

To further confirm BT13 signaling through RET, we assessed RET phosphorylation and activation of its downstream targets in MG87RET fibroblasts expressing GFRα3/RET and GFP/RET. Different doses of BT13 (25–50 μM) elicited phosphorylation of the mature glycosylated form of RET (Figure 1C) represented by a 170 kD band (Runeberg-Roos et al., 2007) and of RET downstream targets, Akt and Erk (Figure 1D)—both in the presence and absence of GFRα3 co-receptor.

**Figure 2:**
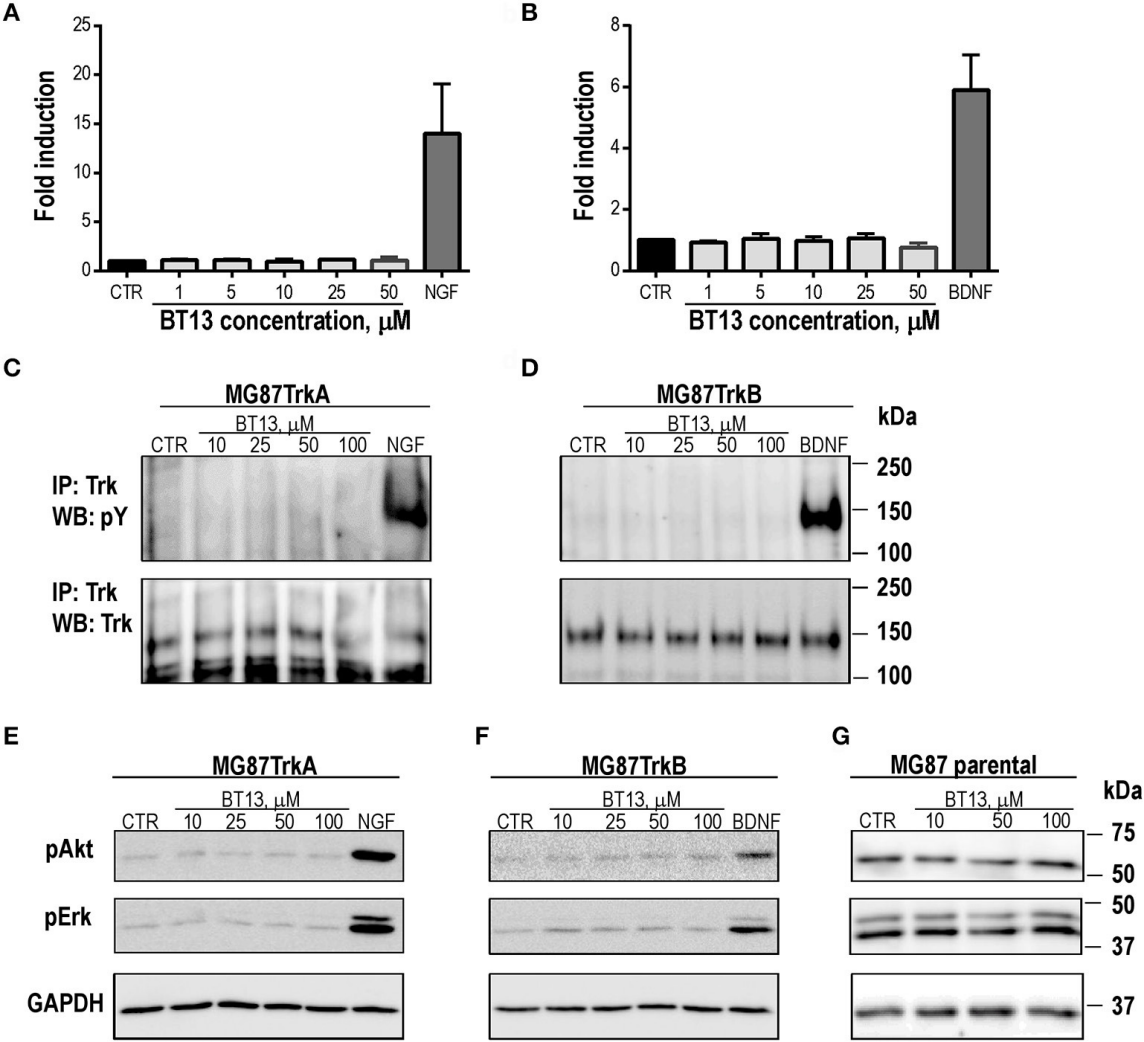
BT13 does not signal in the absence of RET. BT13 does not increase luciferase activity in reporter gene-based assay in cells expressing TrkA **(A)** and TrkB **(B)**. BT13 does not stimulate TrkA **(C)** and TrkB **(D)** phosphorylation. BT13 does not promote activation of intracellular signaling cascades in cells expressing TrkA **(E)**, TrkB **(F)** or parental cells lacking receptors for neurotrophic factors **(G)**. IP, immunoprecipitation; G, GDNF; pY, phosphotyrosine; pAkt, phosphorylated form of Akt; pErk, phosphorylated forms of Erk1/2; GAPDH, glyceraldehyde 3-phosphate dehydrogenase, loading control.

We further studied the specificity of BT13 toward RET using several techniques. First, we assessed whether BT13 was able to activate luciferase reporter gene controlled by the receptors of nerve growth factor (NGF) and brain-derived neurotrophic factor (BDNF), receptor tyrosine kinases TrkA and TrkB, respectively. BT13 did not activate luciferase in either of these two cell lines, although both were fully functional as shown by their responses to the natural ligands NGF and BDNF that were used as positive controls (Figures 2A,B).

To confirm these findings, we assessed phosphorylation of TrkA and TrkB receptors, and the RTK intracellular targets Akt and Erk, in response to BT13 in MG87TrkA, MG87TrkB, and MG87 parental fibroblasts by a direct method (Western blotting). Treatment of MG87TrkA and MG87/TrkB cells with 10–100 μM of BT13 did not produce any detectable increase in the level of TrkA and TrkB phosphorylation, however, these cells responded to NGF and BDNF as expected (Figures 2C,D). In addition, 10–100 μM BT13 failed to significantly stimulate intracellular signaling in the absence of RET as no Akt or Erk phosphorylation was observed after administration of the small molecule in MG87TrkA, MG87TrkB, or parental MG87 cells (Figures 2E–G) in line with the lack of TRK phosphorylation. As expected, NGF and BDNF both promoted Akt and Erk phosphorylation in TrkA and TrkB-expressing MG87 fibroblasts, respectively (Figures 2C–F).

To further validate BT13 as a RET agonist we synthesized derivatives of BT13, which exhibited activity and selectivity identical to the original compound (Bespalov et al., 2016).

### BT13 stimulates elongation and arborization of neurites from cultured peptidergic DRG neurons

To address the putative trophic effects of the RET agonist BT13, we examined neurite outgrowth using adult rat sensory neurons that were isolated from lumbar (L4–L5) DRGs and cultured overnight in defined culture media on a laminin substrate. To increase assay sensitivity, we separately assessed neurite outgrowth in peptidergic C-fiber neurons that are CGRP-immunoreactive (Orozco et al., 2001; Forrest and Keast, 2008; Kalous et al., 2009; Keast et al., 2010; Forrest et al., 2013). These CGRP-positive C-fibers in DRGs express the ARTN receptor, GFRα3, while the CGRP-negative neurons express receptors of other GFLs (Bennett et al., 1998; Senba et al., 2007). Neurite outgrowth was analyzed using two measures: neurite initiation (proportion of neurons that expressed neurites longer than their soma diameter) and neurite elongation (quantified by four morphological parameters).

**Figure 3:**
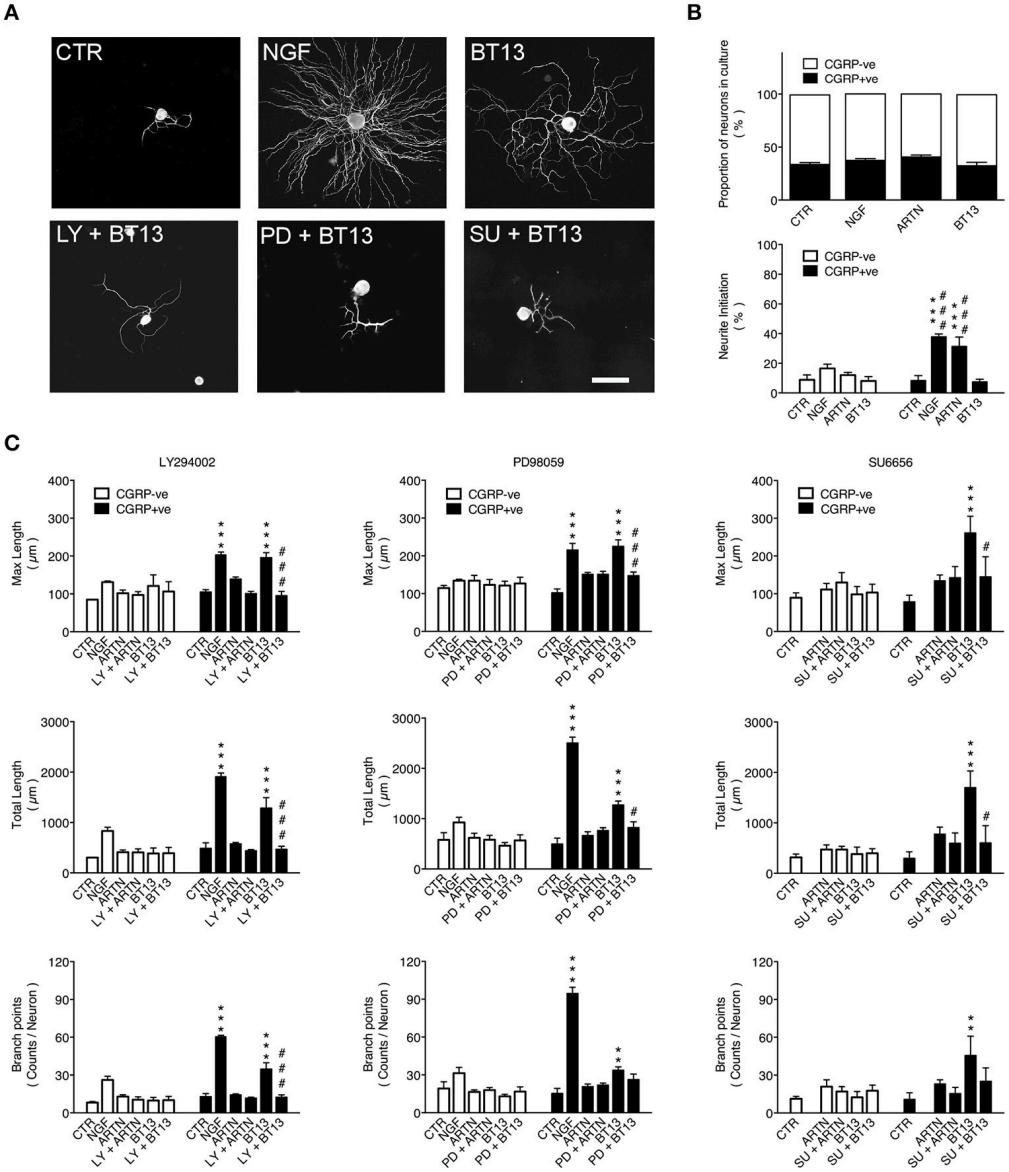
Neurite elongation and branching from peptidergic sensory neurons stimulated by BT13 *in vitro* is blocked by PI3K, MAPK, and Src kinase inhibitors. Neurons were isolated from lumbar (L4–L5) dorsal root ganglia taken from adult rats and cultured for 24 h. Treatment groups were vehicle (CTR), nerve growth factor (NGF: 10 ng/ml), artemin (ARTN, 30 ng/ml), BT13 (100 ng/ml), and either ARTN or BT13 with an inhibitor of PI3K (LY294002, 10 μM), MAPK (PD98059, 50 μM), or Src kinase (SU6656, 2 μM). **(A)** Representative images of βIII-tubulin immunofluorescence in neurons after 24 h in cultures. Image analysis of neurites was used to compare the effects of drug treatment on neurite initiation (proportion of neurons with primary neurites) and elongation (parameters: maximum length, total length, and branch points). Scale bar = 100 μm in all panels. **(B)** Quantification of the percentage of calcitonin gene-related peptide-positive (CGRP+ve) neurons did not detect any change in the proportions of peptidergic (CGRP+ve) and non-peptidergic (CGRP-ve) sensory neurons in cultures treated with NGF, ARTN, or BT13. In the same cultures, quantification of neurite initiation showed significant increases after treatment with NGF or ARTN, but not BT13. ^***^P < 0.05 vs. CTR; ^*###*^*P* < 0.001 vs. BT13, one-way ANOVA with Tukey *post-hoc* test, *n* = 4. **(C)** Quantification of neurite elongation showed all parameters (maximum length, total length, branch points) were significantly increased in peptidergic (CGRP+ve) sensory neurons after treatment with NGF and BT13, but not ARTN. No effect was detected in non-peptidergic (CGRP–ve) sensory neurons. BT13 had no significant effect on maximum length and total length when cultures were also treated with inhibitors of PI3K (LY294002, 10 μM, *n* = 3), MAPK (PD98059, 50 μM, *n* = 4), or Src kinase (SU6656, 2 μM, *n* = *6*). ^**^P < 0.01, ^***^P < 0.001 vs. CTR; ^#^*P* < 0.05, ^*###*^*P* < 0.001 vs. inhibitor, one-way ANOVA with Tukey *post-hoc* test.

Treatments with neurotrophic factor (NGF, ARTN) or BT13 had no effect on neurite initiation or extension in CGRP-negative neurons and did not alter the proportion of DRG neurons that expressed CGRP in culture (Figures 3A,B). In CGRP-positive neurons, neurite initiation was significantly increased by overnight treatment with NGF or ARTN, but not with BT13 (Figures 3A,B). The basal rate of spontaneous neurite initiation in the control group was 8 ± 1% (*n* = 9), which was attributed to the laminin substrate. In accordance with other studies (Wong et al., 2015) NGF and ARTN significantly enhanced neurite initiation, whereas BT13 had no effect on neurite initiation on CGRP-positive neurons (Figure 3B).

Neurite elongation and arborization was assessed using HCA-vision software to analyze neurite morphology (Figures 3A,C) using the parameters: *root number* (number of primary neurites extending from the soma), *maximum length* (length of longest neurite), *branch points* (number of branch points), and *total length* (sum length of all neurites). This analysis confirmed (Lindsay, 1988; Tucker et al., 2006; Wanigasekara and Keast, 2006; Wong et al., 2015) that neurite elongation was significantly increased by NGF (Mean difference: *root number* 3 ± 1, *P* < 0.001; *maximum length* 99 ± 8 μm, *P* < 0.001; *branch points* 48 ± 3, *P* < 0.001; *total length* 1,421 ± 108 μm, *P* < 0.001; *n* = 3) but not ARTN (Figure 3C). BT13 also facilitated neurite elongation as shown by a significant increase in three of the parameters (Mean differences: *maximum length* 91 ± 13 μm, *P* < 0.001; *branch points* 22 ± 5, *P* < 0.001; *total length* 797 ± 207 μm, *P* < 0.001; Figure 3C).

As PI3K, MAPK, and Src kinase are common downstream targets of RET signaling (Airaksinen and Saarma, 2002) we tested if BT13-dependent neurite outgrowth was affected by the specific inhibitors LY294002 (50 μM), PD98059 (50 μM), and SU6656 (2 μM), respectively. The results of these experiments are summarized in Figure 3C. None of the inhibitors had any effect on basal or ARTN-promoted outgrowth, but BT13-stimulated neurite outgrowth was completely blocked by pre-treatment with LY294002 (Mean differences: *maximum length*, 100.7 ± 13 μm, *P* < 0.001; *branch points*, 22 ± 5, *P* < 0.001; *total length*, 817 ± 207 μm, *P* < 0.001), PD98059 (Mean differences: *maximum length*, 78 ± 17 μm, *P* < 0.001; *total length*, 449 ± 113 μm, *P* = 0.031) and SU6656 (Mean differences: *maximum length*, 116 ± 53 μm, *P* = 0.043; *total length*, 1098 ± 343 μm, *P* < 0.001).

### ADME, cytotoxicity, and CEREP profiling

Table 1 summarizes ADME properties of BT13 in biological systems, including: pharmacokinetic parameters, blood-brain-barrier (BBB) penetration, stability in plasma, microsomal stability, human *Ether-à-go-go*-Related Gene (hERG) inhibition and inhibition of major drug-metabolizing forms of CYPs as well as cytotoxicity (Table 1). In rats, BT13 has a Vd equal to 23.55 L/kg, clearance 4.7 L/h and half-life in the blood of 3.47 h. BT13 was stable in plasma but was rapidly degraded by hepatic microsomal enzymes. The fictive distribution volume is such that it readily distributes in tissues and CNS and can easily reach terminal and cell body compartments of sensory neurons (Table 1). This statement is supported by BT13's BBB penetration ratio that was estimated to be 55–68%. Its potential cardiac (assessed by the ability to inhibit human *Ether-à-go-go*-Related Gene, hERG) and hepatic (assessed by cytotoxicity to HepG2 cells) toxicities were found to be low. Finally, BT13 did not interact with CYP1A2, CYP2C9, CYP2C19, CYP2D6, but potently inhibited CYP3A4 *in vitro*, indicating that it might be metabolized by this enzyme (Table 1).

**Table 1:** ADME properties and cytotoxicity of BT13.

**Parameter**	**Species**		
	**Rat**	**Mouse**	**Human**
Vd (L/kg)	23.55	N/D	N/D
Clearance (L/h)	4.7	N/D	N/D
t_1/2_ (h)	3.47	N/D	N/D
Plasma stability (% remaining after 1 h)	108.4 ± 7.3	110.3 ± 3.1	97.0 ± 0.1
Microsomal stability (% remaining in 30 min)	2.8 ± 0.1	N/D	N/D
BBB penetration (%)	55–68%	N/D	N/D
hERG inhibition (Ki, μM)	N/D	N/D	>30
Cytotoxicity to human hepatoma cells (HepG2; LD_50_, μM)	N/D	N/D	90.4 ± 8.5
**INHIBITION OF CYP ISOFORMS (IC_50_, μM)**			
CYP1A2	N/D	N/D	>100
CYP2C9	N/D	N/D	>100
CYP2C19	N/D	N/D	>100
CYP2D6	N/D	N/D	>100
CYP3A4	N/D	N/D	3.2 ± 0.42

*BBB, blood brain barrier; hERG, human Ether-à-go-go-Related Gene; LD50, lethal dose 50, the dose that kills 50% of cells; Ki, constant of inhibition; IC_50_, concentration at 50% inhibition; CYP, cytochrome P450. N/D, not done*.

We assessed the ability of BT13 to interact with selected G-protein coupled receptors, ion channels, transporters and enzymes of neurotransmitter metabolism in the CEREP screen to evaluate selectivity of the compound and potential side-effects. According to the recommendations on the CEREP webpage (http://www.cerep.fr) we set a threshold of 20% for inhibition or potentiation of ligand binding for all tested proteins. Accordingly, BT13 did not interact with any of the tested proteins at 1 μM (Table 2). It is important to note that the concentration of BT13 used in the CEREP screen was approximately five times higher than the concentration eliciting neurite elongation from sensory neurons. These data indicate that BT13 selectively interacts with RET/GFRα and lacks affinity to other studied mechanisms of action.

**Table 2:** CEREP screening of 1 μM BT13.

**Assay (assay type)^a^**	**Cat. No**.	**Percentage of inhibition/potentiation from control**	**Reference compound**	**IC50 Ref (M)**	**Ki Ref (M)**
**G PROTEIN-COUPLED RECEPTORS**					
**Adenosine receptors**					
A1 (h) (antagonist radioligand)	0002	5	DPCPX	8.8E-10	5.6E-10
A2B (h) (antagonist radioligand)	0005	−14	NECA	5.8E-07	5.3E-07
**Adrenergic receptors**					
Alpha 1 (non-selective; antagonist radioligand)	0008	2	Prazosin	4.3E-10	1.1E-10
Alpha 2 (non-selective; antagonist radioligand)	0011	−6	Yohimbine	8.6E-08	3.7E-08
Beta 3 (non-selective; antagonist radioligand)	3963	−5	Alprenolol	1.7E-07	1.3E-07
**Cannabinoid receptors**					
CB1 (h) (agonist radioligand)	0036	9	CP 55940	1.4E-09	1.3E-09
**Cholecystokinin receptors**					
CK1 (CCKA) (h) (agonist radioligand)	0039	3	CCK-8s	8.3E-11	6.2E-11
CCK2 (CCKB) (h) (agonist radioligand)	0041	1	CCK-8s	1.1E-10	4.3E-11
**Dopamine receptors**					
D1 (h) (antagonist radioligand)	0044	−17	SCH 23390	2.9E-10	1.1E-10
D2L (h) (antagonist radioligand)	1405	−3	Butaclamol	1.9E-09	4.9E-10
**GABA receptors**					
GABAB(1b) (h) (antagonist radioligand)	0885	−2	CGP 54626	1.2E-09	5.9E-10
**Histamine receptors**					
H1 (h) (antagonist radioligand)	0870	5	Pyrilamine	1.9E-09	1.2E-09
H2 (h) (antagonist radioligand)	1208	9	Cimetidine	4.6E-07	4.5E-07
**Neurokinin receptors**					
NK1 (h) (agonist radioligand)	0100	−19	[Sar9,Met(O2)11]-SP	3.6E-10	1.6E-10
NK2 (h) (agonist radioligand)	0102	5	[Nleu10]-NKA	2.2E-09	1.2E-09
NK3 (h) (antagonist radioligand)	0104	0	SB 222200	1.2E-08	6.5E-09
**Neurotensin receptors**					
NT (non-selective; agonist radioligand)	0465	−6	Neurotensin	2.9E-09	2.7E-09
**Opioid receptors**					
Delta (DOP) (h) (agonist radioligand)	0114	3	DPDPE	4.1E-09	2.4E-09
Delta (DOP) (agonist effect)	2568	−5.2	DPDPE	1.9E-09	
Kappa (KOP) (agonist radioligand)	1971	0	U 50488	1.2E-09	8.2E-10
Mu (MOP) (h) (agonist radioligand)	0118	1	DAMGO	4.1E-10	1.7E-10
**Vasoactive intestinal peptide receptor**					
PAC1 (PACAP) (h) (agonist radioligand)	1518	14	PACAP1-38	2.2E-10	1.9E-10
**Serotonin receptors**					
5-HT1A (h) (agonist radioligand)	0131	−8	8-OH-DPAT	1.1E-09	6.7E-10
5-HT1D (agonist radioligand)	1974	1	Serotonin	4.4E-09	1.5E-09
5-HT2A (h) (agonist radioligand)	0471	−11	(±)DOI	2.3E-10	1.7E-10
5-HT2C (h) (antagonist radioligand)	0137	11	RS 102221	3.0E-09	9.9E-10
5-HT2C (h) (agonist radioligand)	1003	6	(±)DOI	4.6E-10	4.2E-10
**Ion channels and transporters**					
Ca2+ channel (L, diltiazem site) (benzothiazepines) (antagonist radioligand)	0162	16	Diltiazem	3.3E-08	2.6E-08
KATP channel (antagonist radioligand)	0165	8	Glibenclamide	1.1E-10	3.7E-11
Norepinephrine transporter (h) (antagonist radioligand)	0355	−5	Protriptyline	6.4E-09	4.8E-09
Dopamine transporter (h) (antagonist radioligand)	0052	6	BTCP	1.2E-08	6.5E-09
5-HT transporter (h) (antagonist radioligand)	0439	0	Imipramine	3.2E-09	1.5E-09
**Neurotransmitters synthesis and metabolism**					
MAO-A (antagonist radioligand)	0443	5	Clorgyline	1.3E-09	7.8E-10
COMT (catechol-O-methyl transferase)	0457	−2	Ro 41-0960	2.8E-08	1.6
**Phosphatases**					
Protein Serine/Threonine Phosphatase, PP2A	3953	4	Calyculin A	4.9E-10	1.4
Protein Tyrosine Phosphatase, PTPRC (CD45)	3956	−3	(NH4)6Mo7O24	9.9E-08	1.7

a *All tests were performed in duplicate and average of two repeats is presented in the table*.

### BT13 attenuates mechanical hypersensitivity in animals with experimental neuropathy

We next tested BT13 using a model of experimental neuropathy in rodents. Persistent mechanical hyperalgesia in rats was elicited by ligation of the left L5/L6 spinal nerves (SNL, Chung's model of neuropathic pain). The schedule of compound administration, its duration and delivery method were chosen on the basis of published data for ARTN protein (Gardell et al., 2003) and described in details in Materials and Methods Section.

**Figure 4:**
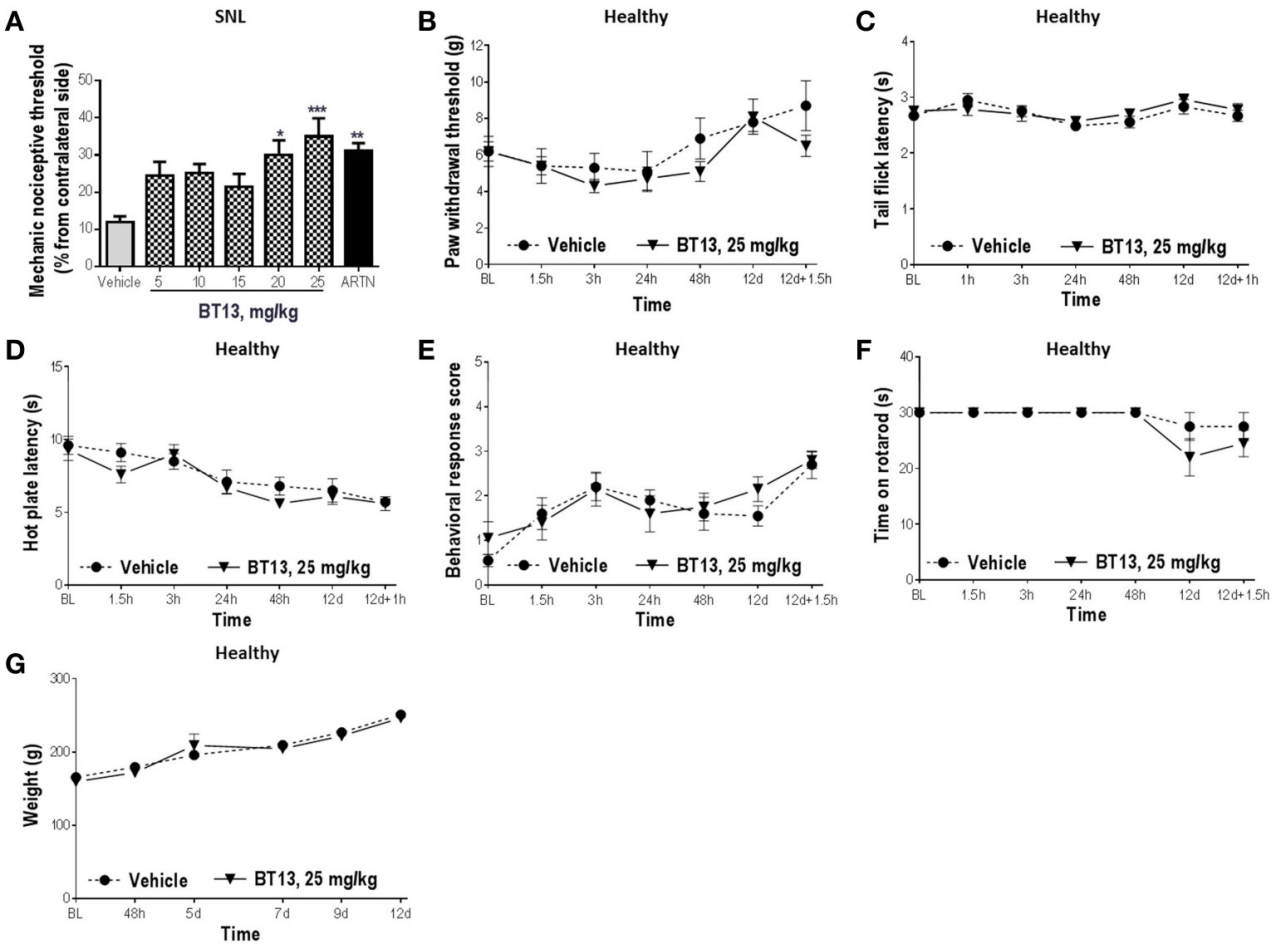
The effects of BT13 in rats with experimental neuropathy and healthy animals. Rats received BT13, ARTN or vehicle subcutaneously once daily on days 1, 3, 5, 8, 10, and 12. Treatments started on day 1 one h after surgery and the last injection of the compounds occurred on day 12 one h before behavioral analysis. BT13 in the doses 20 and 25 mg/kg significantly reduced mechanical hypersensitivity caused by spinal nerve ligation on 12th day post-surgery **(A)**. Thresholds of ipsilateral paws were expressed as percentage from contralateral paw thresholds and used in comparisons between the treatment groups. BT13 did not influence mechanical [paw pressure test **(B)**] or thermal [tail flick **(C)**, hot plate **(D)**, acetone **(E)** tests] sensitivity, motor performance **(F)** and weight gain **(G)** in healthy rats. BL, baseline; ARTN, artemin. ^*^P < 0.05, ^**^P < 0.01, ^***^P < 0.001, ANOVA with Dunnett's *post-hoc* test, *n* = 9–11 **(A)**.

After SNL, rats developed mechanical hyperalgesia as shown by von Frey testing: paw withdrawal thresholds (PWT) of the ipsilateral paws, were significantly lower (8.4 times) compared with the contralateral healthy paws. As expected, a single oral dose of gabapentin (100 mg/kg in water) given on the 12th day post-surgery almost completely abolished mechanical hyperalgesia in rats (24.2 ± 1.83 vs. 26 ± 0.0, ipsilateral vs. contralateral PWT). Repeated post-surgical administration of 20–25 mg/kg of BT13 reduced mechanical hypersensitivity and increased ipsilateral PWTs by 2.5 (*P* = 0.0028, ANOVA with Dunnett's *post-hoc* test) and 2.9 (*P* < 0.0001, ANOVA with Dunnett *post-hoc* test) times, respectively (Figure 4A). Attenuation of mechanical allodynia/hyperalgesia in response to BT13 was comparable with the effect of ARTN which increased ipsilateral PWT by 2.6 times (*P* = 0.001, ANOVA with Dunnett *post-hoc* test). BT13 did not influence contralateral PWTs.

BT13 had no significant effect on baseline sensitivity to mechanical (paw pressure test, Figure 4B) and thermal stimuli (tail flick, hot plate, acetone test; Figures 4C–E) or motor performance (rotarod test, Figure 4F) in healthy animals treated with the compound (*P* > 0.05 for all comparisons, 2-way RM ANOVA). Weight gain in animals treated with BT13 was similar to animals treated with vehicle (Figure 4G). No acute toxic effects, signs of pain or obvious discomfort were observed in experimental animals treated with vehicle or BT13. Significant histopathological changes in major organs (see Section Materials and Methods for details) of animals treated with BT13 were not detected.

### BT13 administration activates intracellular signaling cascades and reduces SNL-induced changes in the expression of neuronal markers in dorsal root ganglia of rats

**Figure 5:**
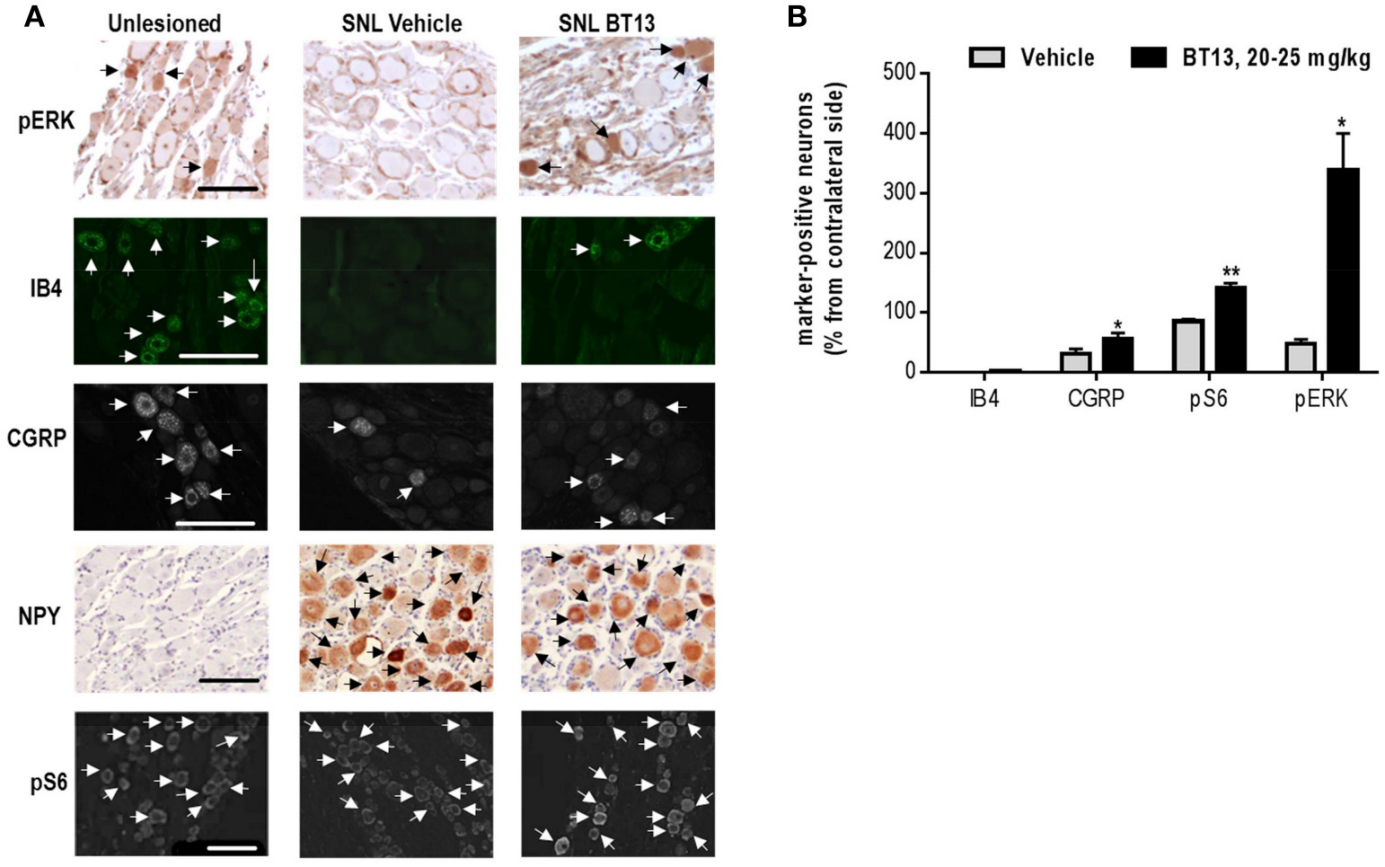
BT13 reverses spinal nerve ligation-induced changes in expression of nociception-related neuronal markers. Samples were collected on 12th day post-surgery after behavioral analysis. Immunohistochemical stainings of DRG paraffin sections with pErk, IB4, CGRP, NpY, pS6 **(A)**. Quantification of IHC data **(B)**. Quantifications of marker-specific immunopositive cells was made for two sections per ganglia, normalized to the total number of PGP9.5-positive cells, averaged, and converted to percentage from contralateral side. Arrows indicate neurons that were counted as marker-specific. The data for 3–6 animals per group were subjected to statistical analysis. Results are presented as mean ± *SEM*. Scale bar—100 μm. ^*^P < 0.05, ^**^P < 0.01, *t*-test.

Since ARTN was shown to restore expression of multiple neuronal markers in damaged sensory neurons (Gardell et al., 2003; Wang et al., 2014), we studied the effects of BT13 on the expression of IB4, CGRP, and NPY in DRG of rats with experimental neuropathy (Figures 5A,B). SNL led to a complete disappearance of IB4-positive and a significant reduction in the number of CGRP-positive neurons in ipsilateral DRGs. NPY expression was not observed in the contralateral ganglia, but after SNL up to 60% of neurons expressed this peptide marker. Administration of BT13 to animals with SNL resulted in detection of a few IB4 positive neurons (in average 3% of IB4 positive cells per section) in ipsilateral DRGs. BT13 also increased the number of CGRP-positive neurons by ~2 times (*P* = 0.047, *n* = 4–6, one-tailed *t*-test) and reduced the number of NPY-positive cells in the ipsilateral DRGs by ~25% (47.64 ± 2.061 vs. 34.77 ± 4.18, *P* = 0.031, *n* = 4, *t*-test). We observed no changes in the numbers of IB4 (44.82 ± 1.960 vs. 44.95 ± 4.295, *P* = 0.978, *N* = 4–6, *t*-test) and NPY (0.0 ± 0.0 vs. 0.0 ± 0.0)—positive neurons, but slight decrease in the number of CGRP-positive neurons (25.09 ± 2.687 vs. 34.30 ± 1.344, *P* = 0.031, *n* = 4–6, *t*-test) in contralateral DRGs of rats treated with BT13 compared to vehicle.

Because our *in vitro* data (Figures 1E, 3C) demonstrated that BT13 activates Akt and Erk signaling cascades in cultures of immortalized murine fibroblasts expressing RET and in primary sensory DRG neurons, we assessed if we could detect their activation *in vivo* using antibodies to phosphorylated Erk1/2 (pErk) and ribosomal protein S6 (pS6) that is downstream target of Akt. BT13 indeed increased the number of neurons positive for pS6 (*P* = 0.003, *n* = 4–5, *t*-test,) and pErk (*P* = 0.011, *n* = 4, *t*-test) in DRGs of animals after spinal nerve ligation in comparison to vehicle-treated animals (Figures 5A,B).

**Figure 6:**
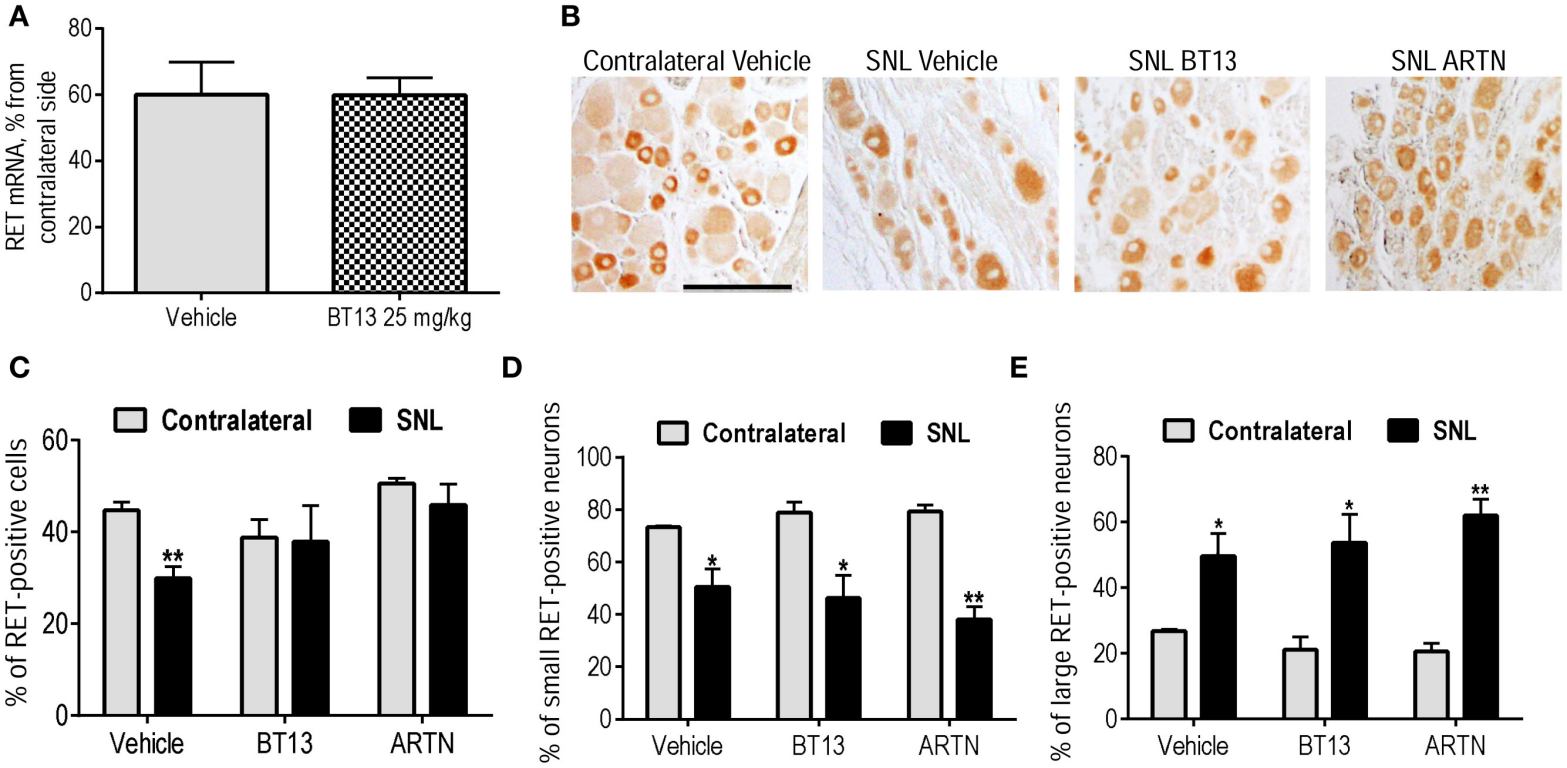
Expression of RET in DRG neurons of animal with experimental neuropathy on 12th day after SNL. mRNA levels of RET in vehicle and BT13-treated SNL animals (in percent from contralateral side) **(A)**. IHC of DRG sections with antibodies against RET **(B)**. Percentage of RET-positive cells in DRGs of rats with experimental neuropathy assessed by IHC: all RET-positive neurons **(C)**, small RET-positive neurons, size below 1,000 μm^2^ **(D)**, large RET-positive neurons, size above 1,000 μm^2^ **(E)**. Two sections per ganglia were quantified and results for 3–4 animals per group were analyzed by *t*-test. Results are presented as mean ± *SEM*. ^*^P < 0.05, ^**^P < 0.01. Scale bar—200 μM.

The major neuronal population expressing RET in rat DRG are IB-4-positive neurons. Minor effect of BT13 on these neurons *in vitro* and *in vivo*, together with its selectivity to RET *in vitro* (Figures 1B–E, 2) was surprising. We hypothesized that the lesion and/or administration of BT13 changed the expression of RET. Therefore, we analyzed the mRNA levels and protein expression of RET in DRGs by qPCR and immunohistochemistry. SNL led to a clear, but not statistically significant trend to reduction in the mRNA level of RET in DRGs that was observed in both vehicle and BT13-treated groups using qPCR (Figure 6A). We also observed a decrease in the number of RET-positive neurons in vehicle (*P* = 0.008, *t*-test), but not BT13 or ARTN-treated animals as shown by IHC (Figures 6B,C). In addition, RET expression pattern in DRG neurons seemed to be altered by SNL. In contralateral DRGs RET is predominantly found in small IB4 positive neurons, whereas in lesioned DRGs of animals treated with vehicle, BT13 or ARTN the number of small RET-positive neurons decreased (*P* = 0.030, *P* = 0.013, *P* = 0.0016, *t*-test, Figures 6B,D) and the number of large RET-positive neurons increased (*P* = 0.030, *P* = 0.013, *P* = 0.0016, *t*-test, Figures 6B,E).

## Discussion

Neuropathic pain caused by peripheral nerve injury affects millions of people in the world but only a minority of patients achieve adequate analgesia with the currently available drugs (Yawn et al., 2009; Jensen et al., 2011; Smith and Torrance, 2012). Here, we identify a candidate small molecule that could be used to reverse chronic somatosensory neuropathology which normally sustains neuropathic pain (Finnerup et al., 2015). Neurotrophic factors ARTN and GDNF hold promise for neuropathic pain management as they can restore damaged sensory neurons (Boucher et al., 2000; Gardell et al., 2003; Wang et al., 2014). However, clinical use of these proteins suffer from complicated delivery to target neurons as they do not penetrate tissue barriers and have high affinity to extracellular matrix (Piltonen et al., 2009; Bespalov et al., 2011). Moreover, large-scale GFL production is very costly which would impact on prescriptions. Thus, small molecular weight mimetics of ARTN and GDNF can be a better alternative to these proteins for therapeutic use.

Attempts to develop molecules activating GFL receptors started more than a decade ago. In 2003, XIB4035 was reported to activate GDNF receptors and elicit downstream effects in cultured cells (Tokugawa et al., 2003). However, it has been recently shown that XIB4035 is not an agonist of GFRα/RET receptors, but instead enhances endogenous or exogenous GDNF and ARTN signaling (Hedstrom et al., 2014). This was sufficient to alleviate diabetes-induced experimental neuropathy by XIB4035. The necessity for GDNF and ARTN may reduce the therapeutic usefulness of XIB4035, as the damage of nerve fibers can limit the availability of GFLs to sensory neurons.

In this study, we characterized BT13, the first small molecule that activates the GFL receptor RET independently of GFLs. *In vitro*, BT13 stimulated phosphorylation of RET, as well as RET-dependent intracellular signaling, but activated neither NGF receptor TrkA nor BDNF receptor TrkB nor intracellular signaling in the absence of RET. BT13 also promoted neurite outgrowth from cultured sensory neurons, had a slight antinociceptive/antihyperalgesic effect and protected DRG neurons in rats with surgery-induced neuropathy.

In contrast to GFLs, BT13 requires no GFRα co-receptors to elicit biological effects (Figures 1B,D). However, it can significantly reduce binding of ^125^I-GDNF to the cells overexpressing GFRα1/RET. Taken together, these data suggest that BT13 can either bind to GDNF receptor RET at the same interface as GDNF [according to SAXS and Cryo-EM model GDNF may interact with CRD domain of RET (Goodman et al., 2014)] or allosterically modulate the affinity of the receptor complex to GDNF. It is generally accepted that RET requires dimerization for activation. BT13 can induce either dimerization of RET via conformational changes or stabilize RET in active conformation, if the preformed RET signaling complex exists (Bespalov and Saarma, 2007). Since GFRα may signal also as soluble protein (Paratcha et al., 2001), we speculate that BT13 mimics soluble GFRα/GFL rather than GFL itself.

In cultured adult DRG neurons, BT13 activated neurite growth only in peptidergic (CGRP-positive) afferents. This class comprises around 20–30% of all DRG neurons and does not express GFRα1 or GFRα2. However, a subset co-expresses GRFα3 with RET and/or the NGF receptors, TrkA and p75 (Orozco et al., 2001; Forrest and Keast, 2008). The effect of BT13 on neurite outgrowth in peptidergic DRG sensory neurons was different from both ARTN and NGF. BT13 failed to stimulate neurite initiation but instead stimulated neurite elongation (Figure 3). This contrasted with lack of BT13 effect on neurite outgrowth from non-peptidergic (IB4 positive) C-fiber afferent neurons, most of which co-express RET in combination with GFRα1 and/or GFRα2 (Molliver et al., 1997; Orozco et al., 2001). In this class of RET-expressing neurons, GDNF increases both neurite initiation and extension but only under permissive conditions when neurons are grown on laminin (Gavazzi et al., 1999; Tucker et al., 2006).

Our results (Figure 3C) suggest that PI3K/Akt signaling is a common target that is required for BT13, ARTN, and NGF to influence neurite outgrowth. However, Src kinase is also required for artemin-induced neurite initiation from peptidergic C-fiber afferents (Wong et al., 2015) and MEK/MAPK is required for GDNF-induced neurite extension from non-peptidergic C-fiber afferents (Tucker et al., 2006). Further study is required to determine if BT13 is effective at stimulating these pathways in isolated primary afferent neurons *in vitro*. The different effects of these agonists on neurons expressing native RET could result from biased agonism that has been most extensively characterized in G protein-coupled receptor signaling systems, but can also occur in tyrosine kinase signaling systems (Kenakin, 2009; Riese, 2011). This is a ligand specific property of receptors that target multiple downstream signaling pathways. In this situation biased agonists can show functional selectivity by activating only a subset of the available downstream effectors. The synthetic GFL receptor agonist BT13 apparently favors a subset of possible GFL signaling cascades which are beneficial to restoring nerve function. An alternate explanation for neurite elongation caused by BT13 in CGRP-positive neurons is non-selective activation of other receptors such as TrkA, but the absence of functional outcomes in our *in vitro* (Figure 2) and *in vivo* (Figures 4B–E) assays suggest that this is unlikely. The lack of effect of BT13 on neurite outgrowth in CGRP negative neurons expressing RET is somewhat surprising and requires further studies.

In line with the results obtained in cultured sensory neurons (Figure 3), in experimental animals BT13 had marked positive effect in CGRP-positive neurons, but mild effect in IB4 positive neurons (Figures 5A,B). In intact rats 20–30% of CGRP-positive neurons express both RET and GFRα3 co-receptors and these are the only DRG neurons responsive to ARTN (Orozco et al., 2001). This is likely the subpopulation of DRG neurons that responded to BT13 treatment in *in vitro* and *in vivo*. To refine our comparison of *in vitro* and *in vivo* effects of BT13 and derivatives, it will also be important to directly compare actions on males and females. To date, we have not identified differences in Trk or GFR expression or responses to neurotrophic factors that would predict a completely different type of action of BT13 in males vs. females, but this does not preclude sex differences in their efficacy, signaling mechanism, or responses to injury.

It is important to note that in surgery-based animal models of neuropathic pain, ARTN treatment positively influenced all types of sensory neurons (Gardell et al., 2003; Wang et al., 2014), even though most classes do not express ARTN receptors. It can be explained by either contribution of less direct mechanisms or by the altered expression of GFL receptors in response to injury or treatment. Indeed, it has been shown that injury affects the expression of GFRα co-receptors in sensory neurons (Bennett et al., 2000; Gardell et al., 2003), and expression of GDNF receptors can be increased in response to GDNF overexpression (Kumar et al., 2015) or lesion (Reeben et al., 1998). We analyzed the expression of RET in DRGs of BT13-treated animals with experimental neuropathy, but observed no difference in the mRNA level of RET in BT13-treated rats in comparison to vehicle-treated rats (Figure 6A). We further assessed the expression and distribution of RET protein in DRG neurons of experimental animals treated with vehicle, BT13, or ARTN. We observed a reduction in the number of RET-positive cells in DRGs of vehicle-treated groups after SNL, restored by both BT13 and ARTN (Figure 6C). Nevertheless, the analysis of the size-dependent distribution of RET in DRG neurons revealed clear changes. In line with previous reports (Bennett et al., 1998; Baudet et al., 2000) in normal DRG, RET was predominantly expressed in small size sensory neurons, but after SNL the proportion of large (>1,000 μm^2^) RET-positive neurons increased (Figure 6E) and the proportion of small RET-positive neurons decreased (Figure 6D). These observations contrast with previous reports that show no changes in RET expression in response to nerve injury (Bennett et al., 2000; Gardell et al., 2003). It is possible that the lesion was more severe in our study, as we observed an ~8-fold decrease in mechanical sensitivity threshold (Figure 4A), whereas in a previously published report sensitivity to mechanical stimuli after SNL decreased by ~4 times (Gardell et al., 2003). In addition, in our study we used an antibody against RET with proven selectivity recently developed by Meka et al. (2015). We were unable to correlate expression of RET and IB-4/CGRP since the expression of both of these markers decreased markedly after the SNL (Figures 5A,B), in line with previously published results (Bennett et al., 1998; Gardell et al., 2003). Nevertheless, it is known that IB4 is mostly expressed in small size DRG neurons and the expression of CGRP is seen in both types. Therefore, it is possible that observed effects of BT13 in different classes of DRG neurons are due to changes in expression and redistribution of RET protein after lesion.

GFLs are survival and maintenance factors for nociceptive neurons and thus the activation of their receptors might provoke pain in healthy animals. Indeed, several studies indicate that overexpression (Elitt et al., 2006) or injections (Lippoldt et al., 2013; Ikeda-Miyagawa et al., 2015) of ARTN and GDNF (Bogen et al., 2008; Hendrich et al., 2012) increased thermal and mechanical sensitivity in rodents. In addition, side-effects of high doses of intravenously administrated ARTN, such as feeling hot, pruritus, and headaches were reported in Phase I clinical trials (Rolan et al., 2015; Okkerse et al., 2016). We tested whether BT13 causes mechanical or thermal hyperalgesia. Administration of BT13 had no effects on pain sensitivity in healthy animals (Figures 4B,D), indicating that (i) its effects are specific to the neuropathic state of the animals and (ii) it may have advantage over ARTN for clinical use. The lack of pronociceptive responses to BT13 can be explained by its selectivity to RET and inability to activate other GFL receptors that can contribute to thermal hyperalgesia (Lippoldt et al., 2013) and/or by biased agonism phenomena that is important for pain-promoting properties of NGF and NGF mutant (Capsoni et al., 2011).

One of the extensively discussed potential side effects of GFL-based medication is related to oncogenic potential of RET receptor tyrosine kinase. Indeed, several mutations in this receptor resulting in its constitutive long-term activation are associated with increased incidence rate of cancers (reviewed in Runeberg-Roos and Saarma, 2007). In addition, ectopic overexpression of GDNF in testicles results in formation of non-metastatic tumors (Meng et al., 2000). However, moderate overexpression of GDNF from its natural locus is not carcinogenic (Kumar et al., 2015). BT13 is a short-living compound having lower efficacy than GDNF (for example its activated luciferase in screening assay by maximum 11.6 fold, whereas GDNF—by 50–100 folds (Sidorova et al., 2010), thus, it is unlikely to promote cancers as a result of RET activation. Indeed, in our experiments we observed no tumor formation in the tissues of rats in response to BT13. However, full spectra of BT13 adverse effects as well as its oncogenic and mutagenic potential are yet to be fully elucidated in further studies.

Taken together, our results suggest that BT13 is a novel selective RET agonist that could lead to further development of novel treatments for neuropathic pain. In contrast to GFLs, its production and administration is easier, and it may have fewer side effects due to its selectivity for RET and/or biased agonism phenomena, when compared to the endogenous neurotrophic factors. BT13 itself has a modest antihyperalgesic effect and we are currently optimizing its potency and efficacy as well as pharmacological characteristics using methods of computational and medicinal chemistry. In addition, we plan to improve the delivery strategy and administration schedule for RET agonists.

## Author contributions

YS, AW, OK, PR, PO, JK, EK, MK, and MS contributed to the design of the work, acquisition, analysis, interpretation of the data, drafting the manuscript; MB has discovered, validated and performed initial characterization of BT13 family of compounds, contributed to the design of the work, interpretation of the data, revising manuscript critically for important intellectual content; VJ and TL contributed to the acquisition, analysis, interpretation of the data, drafting the manuscript; IS and GK contributed to the acquisition and analysis of the data. All authors approved the final version of the manuscript to be submitted.

### Conflict of interest statement

MB, MK, and MS are inventors in composition of matter patent on of BT compounds US 8,901,129 B2. MK and MS are inventors in patent application WO 2014041179 A1. The present study was partially supported by GeneCode Ltd. The other authors declare that the research was conducted in the absence of any commercial or financial relationships that could be construed as a potential conflict of interest.
